# Saphenous nerve injury in ACL reconstruction: higher risk with dual hamstring harvest – an observational study

**DOI:** 10.1051/sicotj/2026051

**Published:** 2026-10-15

**Authors:** Khayas Omer Kunheen, Vishnu Subramaniyan, Raju Karuppal, Sibin Surendran, Jijulal Cu, Ramesh Govindharaaju

**Affiliations:** 1 Department of Orthopaedics, Government Medical College Kozhikode Kerala India; 2 Sports Injury Centre, VMMC & Safdarjung Hospital New Delhi India; 3 Department of Orthopaedics, KMCT Medical College Hospital Mukkam, Kozhikode Kerala India

**Keywords:** ACL, Saphenous nerve, Infrapatellar branch, Sartorial branch, Hamstrings, Tendon stripping, Sensory disturbance

## Abstract

*Introduction*: Injury to the saphenous nerve or its branches is one of the most common complications of Arthroscopic ACL reconstruction surgery. Among the various iatrogenic reasons for this injury, nerve injury during the passage of the tendon stripper (due to the anatomical proximity of the nerve to the tendons) has been reported already. This study compares the incidence of the saphenous nerve injury following isolated semitendinosus graft harvest versus combined semitendinosus and gracilis harvest during Arthroscopic ACL reconstruction. *Methods*: A prospective observational study of 120 patients, who had undergone arthroscopic ACL reconstruction with semitendinosus graft alone (60 patients) and semitendinosus + gracilis graft (60 patients) from April 2020 to September 2023 was conducted. A follow-up regarding the presence of sensory changes in the saphenous nerve distribution was done at six months. *Results*: 28.3% (17/60) of patients in the semitendinosus group (group A) had sustained sensory disturbances compared to 53.3% (32/60) of patients in the semitendinosus + gracilis group (group B). This difference was found to be statistically significant. However, the nerve injury did not interfere with the daily activities of the majority of patients. *Conclusions*: The saphenous nerve is at a higher risk of injury during double hamstring graft harvesting. Surgeons may avoid resorting to double hamstring harvest whenever possible. Tripled or quadrupled semitendinosus is a viable option in most situations. Techniques like All inside ACLR with shorter graft lengths are worth considering in this context.

## Introduction

Anterior cruciate ligament reconstruction (ACLR) using hamstring tendons is one of the most popular arthroscopic surgeries performed all around the world. Grafts prepared from the tendons of the gracilis and semitendinosus (ST) muscles are increasingly being used because these grafts withstand high loads before failure; their cross-sectional area is large; they pass easily through the tunnels; and they only need a small incision for harvesting and give rise to lower morbidity at the donor site [1]. However, because of their anatomical location, there is a potential risk of injury to the infrapatellar and sartorial branches of the saphenous nerve (SN) during the harvesting of these tendons [2–4].

For meeting patient expectations, harvesting techniques for autogenous hamstrings are becoming increasingly minimally invasive. As a result, single skin incision of 3 cm or less and distal-to-proximal tendon harvesting for hamstring autografts are gaining popularity. However, graft harvesting is not free of risks. The commonest complication is anterior tibial hypoesthesia, neuropathic pain, or painful neuroma caused by injury to the saphenous nerve or its branches. Disruption of the saphenous nerve at the knee may cause a spectrum of neuropathy ranging from insignificant sensory loss to painful neuralgia variably described by patients as burning or electrical shock-like sensations [5–7]. With increasing emphasis on limited incisions, accurate knowledge of local anatomy is paramount to avoid iatrogenic neurologic injury while at the same time ensuring a complete harvest of the ST and gracilis. In the literature, the incidence of saphenous nerve injury during autologous hamstring harvesting for ACLR is upto 74% [8] to 88% [9]. The anatomy of the saphenous nerve and its branches has been extensively studied in cadaveric dissections by various authors in the past [4, 10, 11] (Figure 1). The saphenous nerve has only sensory nerve fibres which innervate the superficial sensation of the medial knee, which includes parts of the anterior patella, medial tibial crest, posterior medial leg, and medial foot. But this innervation is complex, and several anatomical and clinical studies have proposed varying dermatomal patterns [1, 4, 8, 11–13] (Figure 2). The saphenous nerve originates from the posterior branch of the femoral nerve in the proximal femur and enters the adductor canal on the medial side. It divides into two mostly identifiable terminal branches: the infrapatellar branch (IPBSN) and the sartorius branch of the saphenous nerve (SBSN) (Figures 1 and 3). The IPBSN then divides mostly into two more branches (up to four branches described) – the upper and lower branches, which innervate the anterior skin of the knee around and below the infrapatellar region. The location of the IPBSN varies across individuals and even between the two lower limbs of the same individual [4]. It has been reported that the IPBSN can be damaged by minimally invasive incisions like arthroscopic portals, hamstring harvest incision, bone patellar tendon bone (BPTB) graft harvest, or even by blunt contusion from a hamstring tendon-stripping device. Injury to the IPBSN results in the development of sensory disturbance from the anterior knee to the proximal lower leg (Figures 2 and 4). This injury requires attention because it occasionally gives rise to anterior knee pain, kneeling pain, painful neuroma, and CRPS [5, 6]. The sartorial branch sensory disturbances are comparatively very well tolerated by patients in general due to the anatomic location of its supply, which is in the anteromedial leg distally (Figure 4). The incidence of IPBSN injury has a direct and very important relationship with the type of incisions placed during arthroscopic ACLR [14]. The sartorial branch takes a more vertical course alongside the saphenous vein distally as it goes down the medial knee to provide sensation to the medial aspect of the leg, ankle, and foot [15] (Figure 1).

**Figure 1:**
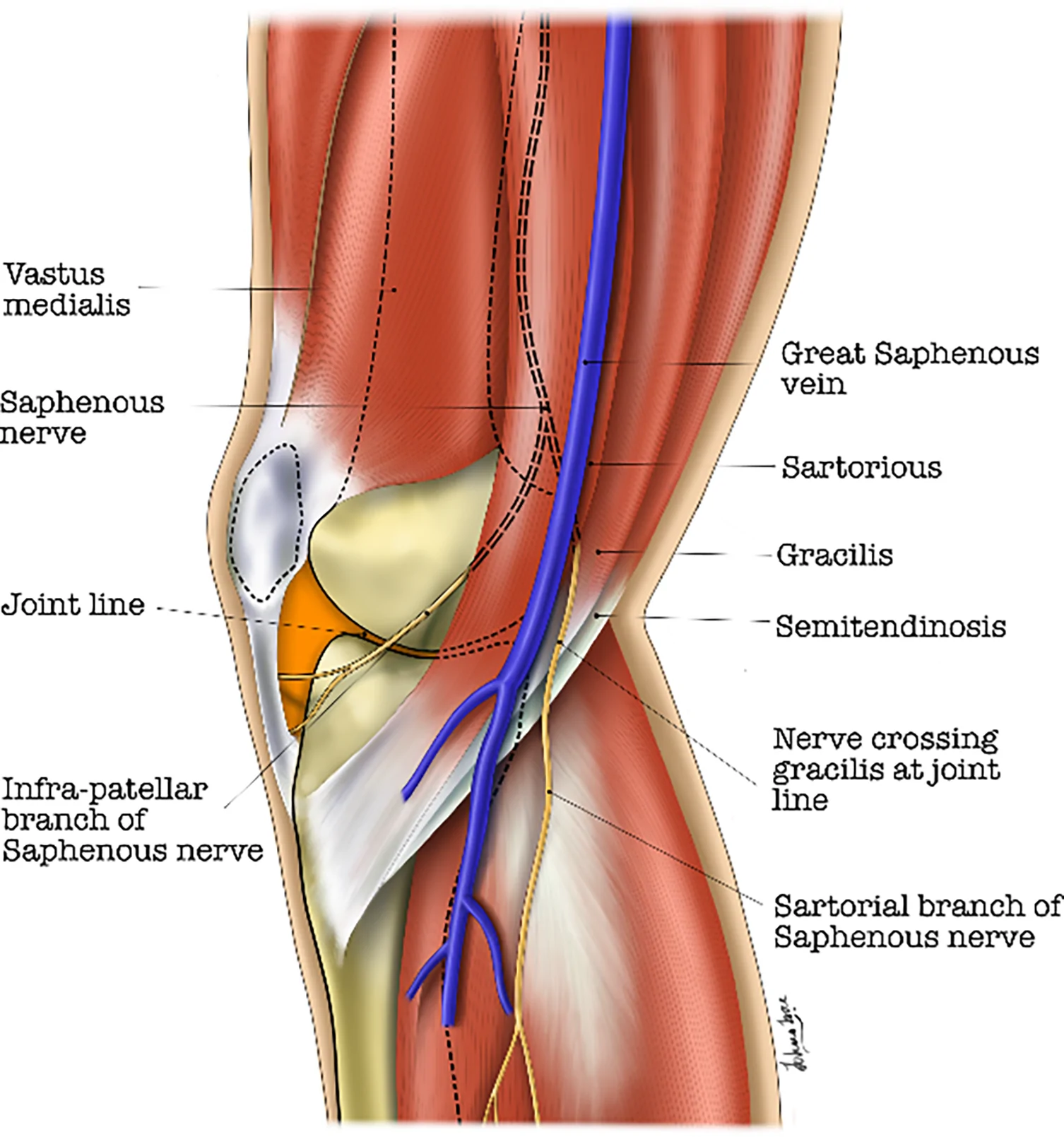
Schematic diagram showing course and relations of Saphenous nerve and its branches.

**Figure 2:**
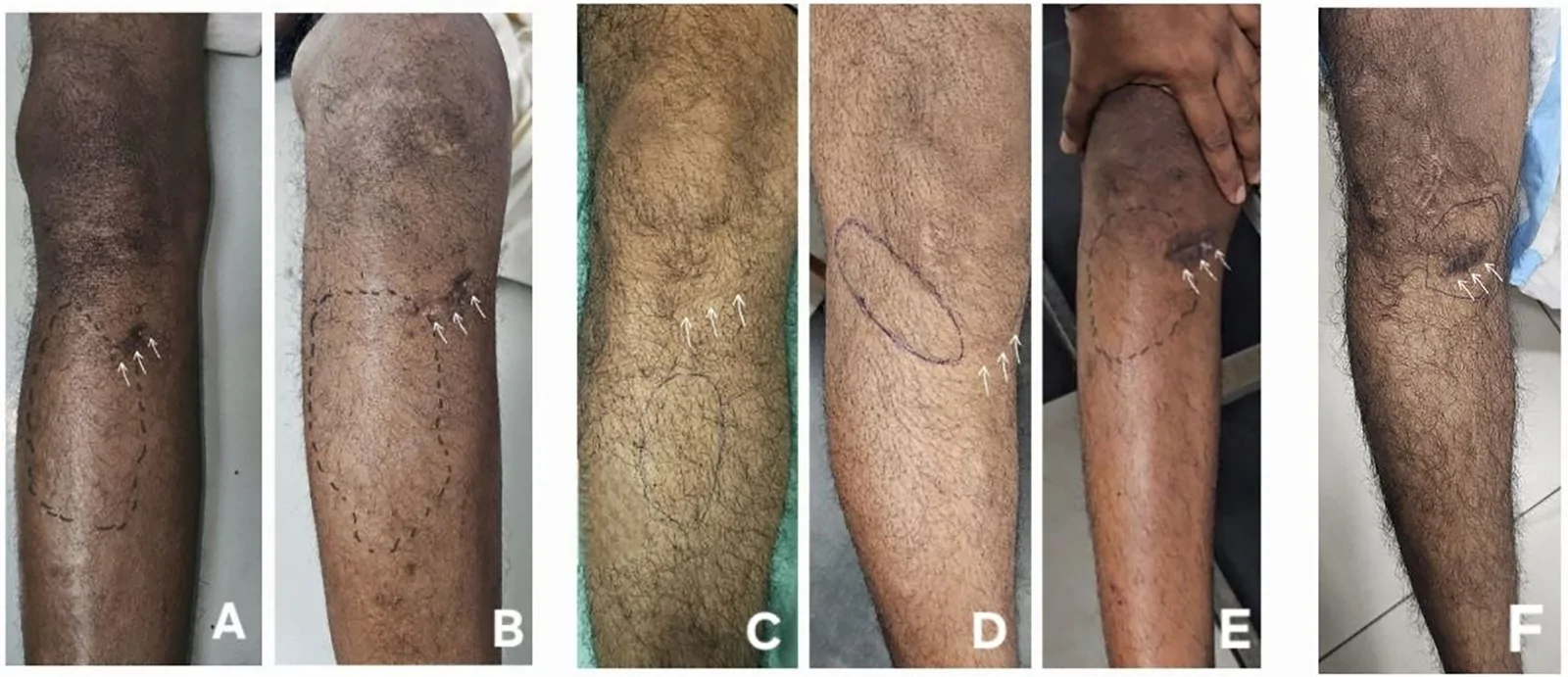
Clinical photographs denoting areas of sensory disturbances as described by various patients on follow-up. Shows right knees with varying dermatomal patterns of injury to IPBSN (Patient D, F), SBSN (Patient B, C), and combined IPBSN and SBSN (Patient A, E). White arrows highlighting graft harvest incision.

**Figure 3:**
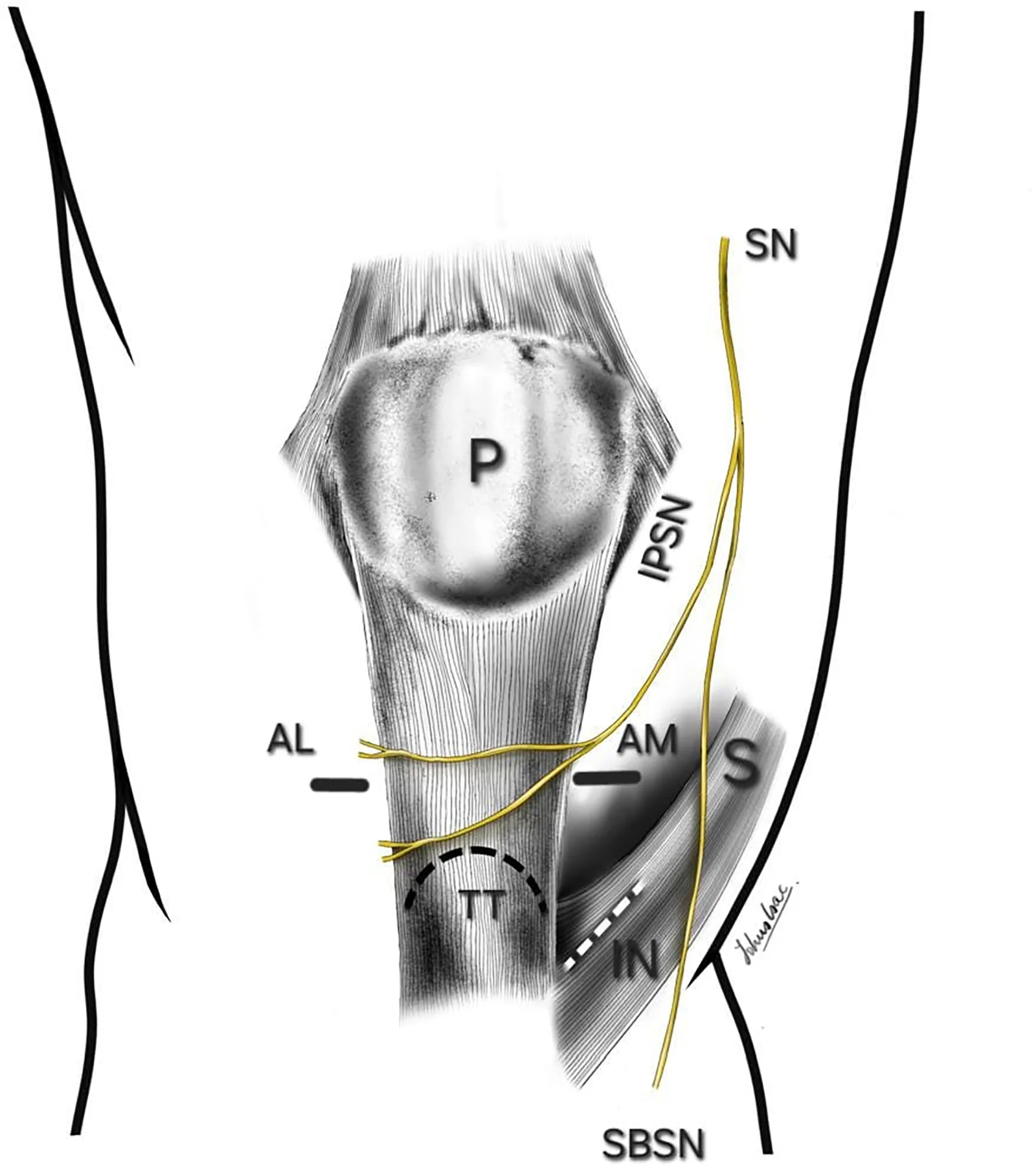
Relation of saphenous nerve and its branches to the standard portals and harvest incision in ACLR. AL-anterolateral portal, AM- anteromedial portal, TT-tibial tubercle, P-patella, SN-Saphenous nerve, IPBSN-infrapatellar branch, SBSN-sartorial branch, S-sartorius muscle, IN-Graft harvest incision.

**Figure 4:**
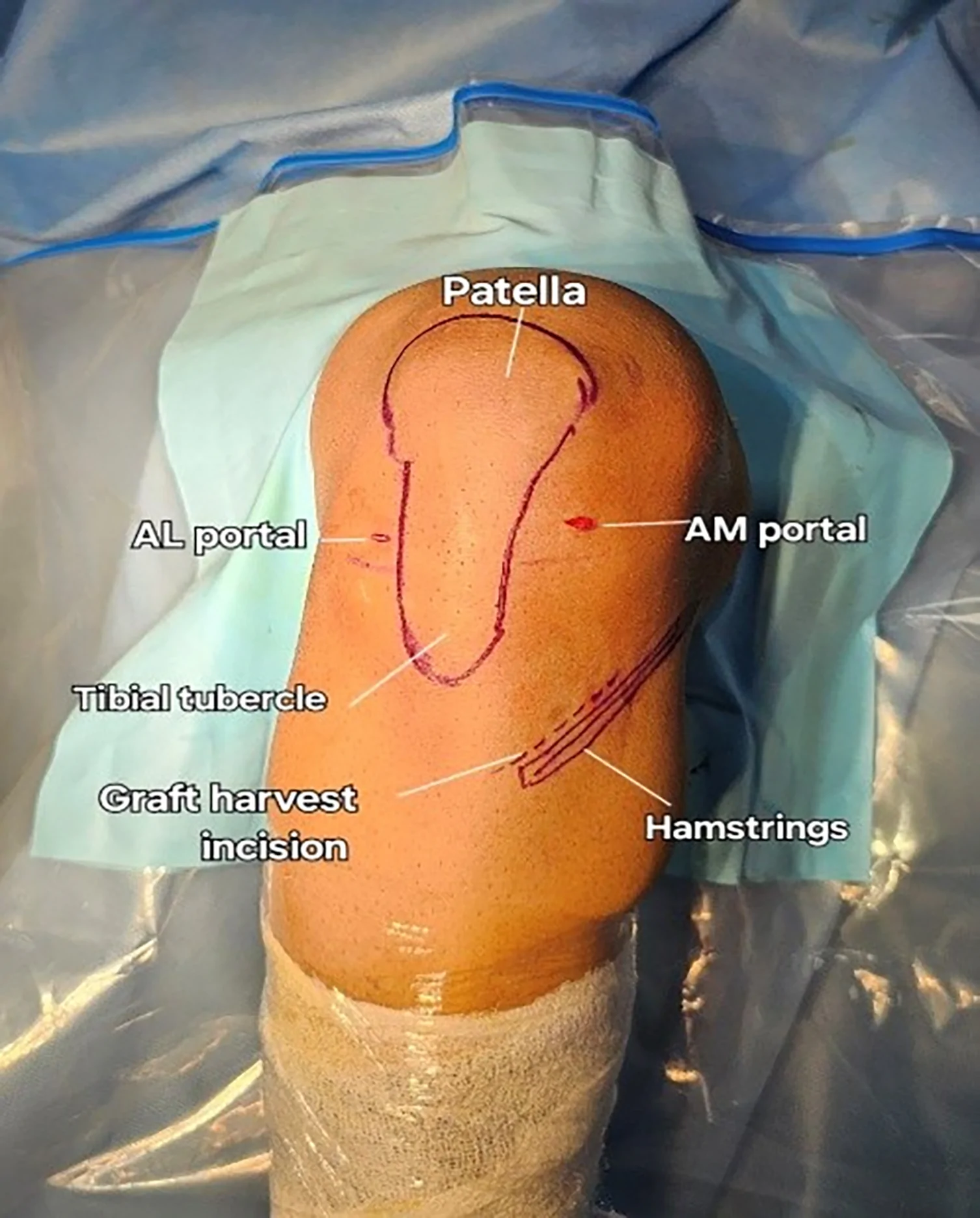
Schematic diagram used to categorise patients. Shows two major cutaneous distributions of the saphenous nerve (SN). IPBSN-Infrapatellar branch of SN. SBSN – Sartorial branch of SN.

Various attempts have been made to correlate the sensory disturbances in the saphenous nerve dermatome with the surgical steps leading to the same. Arthroscopic ACLR using standard two portals and hamstring autograft harvest using a single medial incision has been considered the standard in most of these studies (Figure 5). The portal incision, graft harvest incision (its location, angle, size), knee position during graft harvest, etc., have been variables under consideration affecting the outcome [2, 4, 8, 11–13, 16, 17]. This type of injury has also been reported in medial meniscal repairs (especially inside-out and outside-in techniques), posteromedial portals, and open medial procedures [5, 8, 18]. Only a few authors have focused on the sartorial branch and studied the possibilities of injury to the same during ACLR, especially during steps like hamstring tendon stripping in a distal to proximal fashion [3, 5, 7, 8, 14, 18]. We presume harvesting the hamstring with a tendon stripper is a potential threat to the saphenous nerve (or sartorial branch according to the level of bifurcation) as it crosses the posterior part of the gracilis tendon at the posteromedial joint line (Figure 1) [4]. We hypothesise that harvesting semitendinosus alone may have a better prognosis in terms of sensory disturbances compared to combined gracilis + ST harvest. Not many authors have studied this, probably, and no one has reported this from Asia to the best of our knowledge [7].

**Figure 5:**
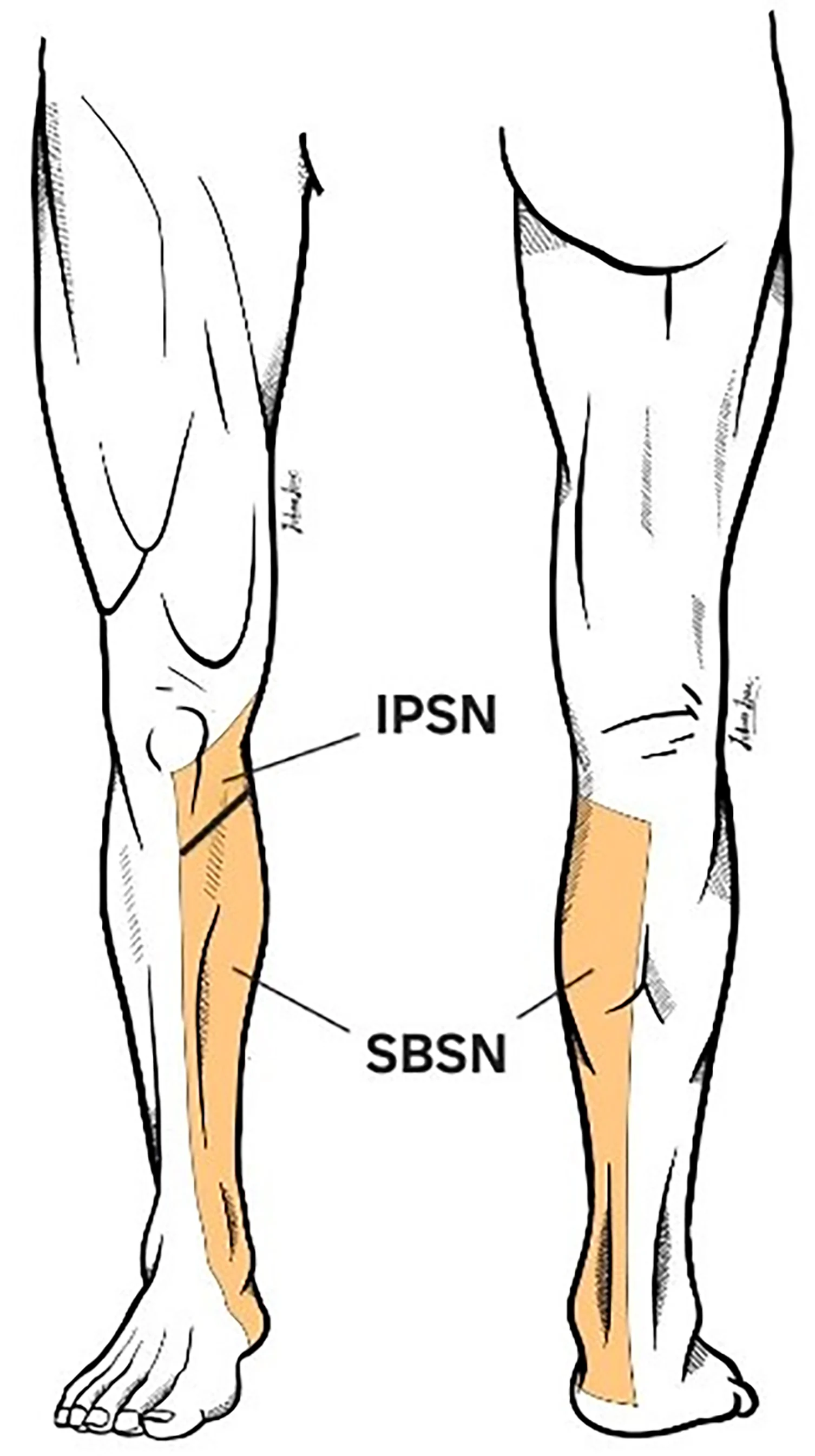
Intraoperative image showing standard horizontal portals and graft harvest incision.

Recently, more and more surgeons consider it adequate when they have a triple or more commonly quadruple isolated ST graft, provided they get a girth of at least 8 mm. It has been published that a minimum of 15 mm of graft length is adequate inside the femoral tunnel [19], and on many occasions, the isolated ST graft meets the girth and length requirements of the ideal graft. We hypothesise better outcome with an isolated semitendinosus harvest in terms of sensory disturbances by safeguarding the gracilis. This can be considered an added advantage, apart from the benefit of a retained hamstring structure (which in turn facilitates regrowth of harvested semitendinosus) and, in turn, better knee flexion power recovery [20].

With the evolution of surgical procedures for ACL reconstruction, patients’ expectations regarding the results are growing. They seek to return to their daily activities earlier, and that too with less morbidity. In the present circumstances, our study is relevant as it helps surgeons to find a way to reduce the incidence of saphenous nerve injury, which is one of the most common complications following ACL reconstruction.

## Materials and methods

After institutional ethics committee approval and informed consent from the participants, a prospective observational study was conducted in a convenience sample of 120 patients who presented to the Orthopedics department at Government Medical College, Kottayam, Kerala, India, with ACL injury requiring arthroscopic reconstruction using hamstring autograft, during the period of April 2020 to September 2023. Patients with meniscal injuries that required inside-out or outside-in repair, associated ligament injuries that required operative intervention, previous knee surgeries, and spine-related radiculopathies or other pre-existing neuropathies affecting the knee or leg were excluded from the investigation.

A single surgeon performed the ACL reconstructions using standard AM and AL portals without many variations in the technique over the study period. The tourniquet used was inflated to 280 mmHg and deflated within 1.5 h in all patients. Great attention to minimise preventable reasons for SN injury was undertaken, and every attempt was made to curtail inadvertent SN injury (as enumerated hereafter), thereby reducing confounding errors meanwhile strengthening the results of our study. Horizontal incisions were used for portals [11, 17]. One was made in the lateral infrapatellar area in the soft spot 1.5 cm above the joint line (Figures 3 and 5). The second was made at the medial infrapatellar area, 1.5 cm above the joint line for use as a working portal. After arthroscopic surgery, the skin was sutured with a single 3-0 nylon thread stitch. The harvesting of the graft, including the release of the vincula, was done under good visual control in a blunt manner. The stripper had a blunt and round distal edge. When suturing, only the skin was closed, and the subcutaneous tissue and fascia were left open. Cases with associated meniscal injuries requiring meniscal balancing and all inside meniscal repair were not excluded from the study. This was based on a recommendation by Koch et al., who proposed a higher incidence of SN injury in inside-out or outside-in repair [21].

An Oblique incision (around 45°) was placed in our cases as per the recommendation by Boon et al. [2, 22] to minimise the inadvertent IPBSN injuries due to the vertical incision. It was placed following the rule of two proposed by Jiang et al. [10], i.e., the centre of the 3 cm long incision was 2 cm distal and 2 cm medial to the tibial tuberosity (TT). The sartorius fascia covering the hamstring tendons was opened along the direction of tendons using scissors, and with the help of right-angle forceps, the ST was isolated and harvested using an open stripper (pig tail type), maintaining its distal insertion in the tibia. Stripper was used in a distal-to-proximal fashion to harvest ST after releasing all the fascial bands to the surrounding myofascial structures under vision. A knee in flexed position with hip in external rotation in a figure of 4 position was used for tendon harvesting [4, 17]. The distal footprint was carefully dissected. At the conjoined part of the pes insertion, ST was separated from the gracilis with a sharp knife, assisted by traction applied over the severed ST. Finally, the ST was separated from the bone, making sure the maximum amount of periosteum is also harvested, increasing the prospect of avoiding gracilis harvest by getting an extra inch or more length of ST.

The decision to undertake harvesting both ST and gracilis or ST alone was taken intraoperatively, solely depending on the length and girth of the available ST. A girth of 8 mm or more of harvested ST on quadrupling was considered ideal for the procedure. Overall graft length of 70 mm with a minimum of 15 mm in the femoral tunnel was deemed adequate. The rest of the cases with inadequate graft dimensions had gracilis too harvested. In this way, selection bias was avoided. Suspensory button loop fixation was used on the femoral side, and titanium interference screw fixation on the tibial side. Gracilis, when harvested, was used to build a 5-stranded or 6-stranded final graft. Patients were dichotomised into two groups based on the tendons harvested. The study was to be continued for 3.5 years until 60 patients were recruited in each group and completed a 6-month follow-up, which was deemed a necessary sample size for statistical significance. A few patients who did not turn up for follow-up were omitted, and new patients were recruited in their place. No drains were used. Standard postoperative protocol was followed. Patients were evaluated for the study six months postoperatively and were given a questionnaire to fill out basic details and other information, like whether these symptoms were serious enough to affect their daily affairs. They were asked to draw over their legs using a skin pencil, the areas of the skin with altered sensations (Figure 2). Based on these drawings, a schematic diagram of the cutaneous sensory supply of the IPBSN and the SBSN (Figure 4) was used by the evaluators, who were blinded as to the details of the graft harvested, to categorise the patients into three groups – 1) altered sensation only in the IPBSN distribution, 2) only in the SBSN distribution, and 3) in both these distributions. If the patients had sensory abnormalities in regions outside the distributions shown in the schematic diagram (especially lateral to midline), the following rule was followed. All findings proximal to the level of graft harvest incision were taken as IPBSN affection and below the incision as SBSN affection (Figure 2) 12 patients who were not available for physical follow-up were contacted over the phone, and 4 out of them had sensory disturbances. They were asked to fill out the proforma and mail the picture of their sensory markings back to us.

Statistical analyses were conducted using the Statistical Package for the Social Sciences (SPSS), version 18.0 (IBM Corp., Armonk, NY, USA). Continuous variables were summarised as mean ± standard deviation, while categorical variables were expressed as frequencies and percentages. Group-wise comparisons of continuous outcomes were performed using the unpaired *t*-test, and categorical data were analysed using the chi-square test. To assess changes in outcomes over time, repeated-measures analysis of variance (ANOVA) was employed, with within-group comparisons evaluated using paired *t*-tests where appropriate. Effect sizes were calculated to estimate the magnitude of between-group differences, and 95% confidence intervals were reported for key outcomes to enhance clinical interpretability.

Baseline demographic and clinical variables, including age, sex, side of surgery, and graft dimensions, were comparable between groups. Surgical technique, incision placement, and tendon harvesting method were standardised and performed by a single surgeon, thereby minimising procedural variability. Given the balanced baseline characteristics and limited event rates, multivariable adjustment was not undertaken. A *p*-value <0.05 was considered statistically significant.

## Results

Table 1 shows the proportion of patients in each group who had saphenous nerve injuries. Table 2 explains the proportion of the branches of the saphenous nerve injured in each group. 87.5% patients (105 out of 120) were in the age group 20–40 years. 80% were males (96 out of 120). Sensory disturbance was seen in 49 out of 120 (40.8%) patients at the end of six months of follow-up. 28.3% (17/60) of patients in the semitendinosus group (group A) had sustained sensory disturbances, compared with 53.3% (32/60) of patients in the semitendinosus + gracilis group (group B). The chi-square test comparing this result was highly significant (*p* = 0.005), indicating that the occurrence of postoperative altered sensitivity is higher with the harvesting of two tendons.

**Table 1:** Proportion of patients with SN injury in each group.

Group		Altered sensitivity	Normal sensitivity	Total	χ^2^	*P*-value
**A** (*Semitendinosus alone*)	Count	17	43	60		
	%	28.3%	71.7%	100%		
**B** (*Semitendinosus + Gracilis*)	Count	32	28	60		
	%	53.3%	46.7%	100%	7.76	0.005
Total	Count	49	71	120		
	%	40.8%	59.2%	100%		

**Table 2:** Percentage of nerve injury according to the branch involved at six months.

Group		Branches involved at 6 months			Total
		IPBSN Alone	SBSN Alone	IPBSN + SBSN	
**A** (*Semitendinosus alone*)	Cases	5	2	10	17 out of 60
	%	8%	3%	17%	
**B** (*Semitendinosus + Gracilis*)	Cases	7	5	20	32 out of 60
	%	12%	8%	33%	

Total out of 120		12(10%)	7(5.8%)	30(25%)	49 out of 120

In the questionnaire, only a mere 7.5% (9 out of 120) patients reported that the numbness or paraesthesia was significant enough to interfere with their daily activities. Apart from the sensory disturbance, one patient from group B had developed joint infection, two from each group had developed superficial graft site infection, and one from group B had developed deep graft site infection. In the ST group, one had developed knee stiffness, while in the other group, three had developed stiffness (not affecting their daily function) at 6 months follow-up. None of the patients in both groups developed painful neuroma or complex regional pain syndrome.

## Discussion

Arthroscopic ACL reconstruction is reported to have several complications, among which saphenous nerve sensory disturbances are one of the most common. Our study was aimed at understanding one of the factors that may lead to altered sensations, which was decided to be tendon stripping. In the present study, we attempted to find out the incidence of nerve injury associated with harvesting of semitendinosus alone and combined semitendinosus + gracilis tendons during ACL reconstruction.

At 6 months follow-up, our results showed a statistically significant difference between the incidence of sensory alterations between the two groups 28.3% in group A versus 53.3% in group B (Table 1). This result goes in accordance with the findings of Padua et al. [7], where they reported 36.1% in the ST group versus 58.1% in the combined group. The total proportion of patients with sensory affection in our study was 40.8% (49/120), which was also comparable to previous reports [7, 10, 11, 18]. Out of this 40.8%, on 6 months follow-up, 10% patients had sensory alterations in the IPBSN dermatome alone, 5.8% had in the SBSN dermatome alone, and 25% patients had IPBSN and SBSN combined dermatome affection (Figure 2) (Table 2). Several authors have reported IPBSN involvement from 32% [23] up to 72% [3]. Our low incidence (10%; 12 out of 120) may be due to the extremely careful steps we followed as described earlier in the methods section to minimise IPBSN injury.

Pagnani et al. and Arthornthurasook et al. in their anatomic study [4, 15] on cadavers evidently described the intimate relation of the saphenous nerve with the gracilis tendon. Soon after exiting the adductor canal, the infrapatellar branch is given at a region proximal to the point at which the nerve crosses the gracilis tendon at the posteromedial joint line [4]. Variations of this branching have been reported by Koch et al., who showed 15% cases with branching below the joint line [24] (Figure 6). After giving the IPBSN branch (which goes anterior to, posterior to, or through the sartorius) [15], SN goes vertically down as SBSN beneath the sartorius and pierces the fascia between the sartorius and gracilis to exit superficially [15] and then accompanies the saphenous vein, crosses posterior to the gracilis at the joint line in the subcutaneous plane to supply the medial leg and ankle. Here at the posteromedial joint line, it is seen consistently lying immediately superficial and posterior to the gracilis tendon (Figure 1). Pagnani and Tifford et al. [4, 17] even reported that the knee in the flexed position (with hip in external rotation in a figure of 4 position) is safer than the extended position for tendon harvesting, just because the tension on the nerve increases in extension, pulling it more towards the gracilis belly. Our hypothesis of stripper related injury was based on this anatomical fact, and our results show that this knowledge will be handy for the surgeon to reduce the incidence of postoperative saphenous sensory disturbance by resorting to isolated ST harvest, safeguarding the gracilis. Retained gracilis also may enhance ST regrowth as suggested in some studies [20].

**Figure 6:**
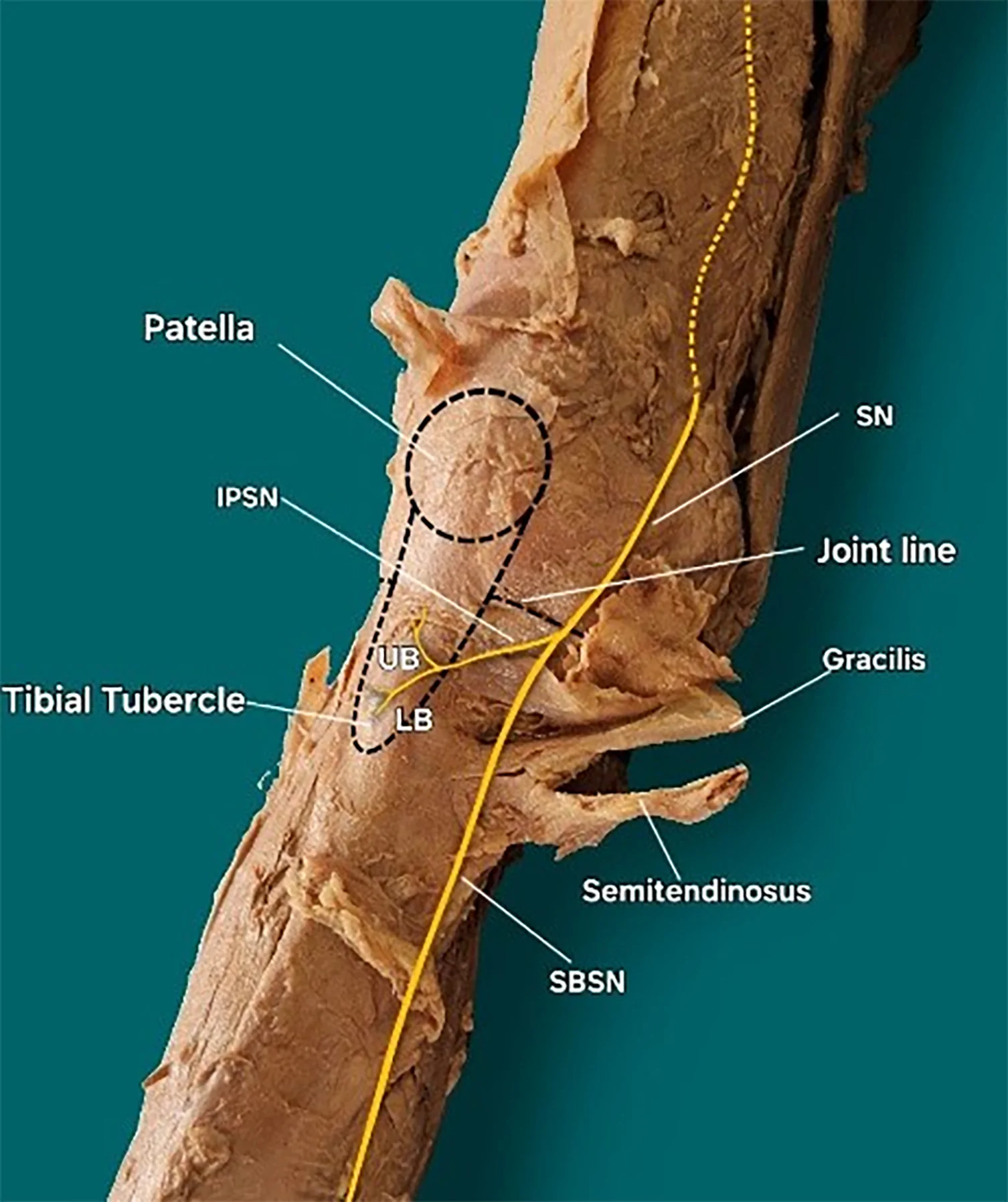
Dissection specimen with schematic representation of Saphenous nerve (SN) showing infrapatellar nerve (IPBSN) branching below the joint line. UB – upper branch, LB – lower branch.

Dunaway has reported a more common variation whereby SBSN will be deep to the fascia at the joint line [18]. Also, he stated SBSN was never seen to stay posterior or lateral to the ST, unlike gracilis, which has a close relation to the SBSN, which again proved our hypothesis of safe ST alone harvest. SN branching can happen near the joint line, above it, or below it (Figures 1 and 6) [24]. This can explain the SBSN alone affection in our series (although only 6% cases) according to the level of bifurcation. Dunaway has reported that at the joint line, during procedures like meniscal repair, where you venture posterior to sartorius, there is a greater chance for SBSN alone rather than IPBSN injury, which again proves the early bifurcation theory.

We used the rule of two for placing the oblique incision for graft harvesting [10]. Pagnani, in his elaborate anatomical manuscript, localised the pes tendons to be usually around 2 cm below the tibial tuberosity. Kerver et al. [13] suggested that there is only a meagre possibility of IPBSN to course distal to the tibial tuberosity. Ebraheim, Tifford and Mochida also reported similar findings [11, 16, 17]. So, we presume our tendon harvesting incision, causing IPBSN injury, is very unlikely.

Mirzatolooei et al. [3] reported that 9.8% of the patients who had nerve injuries had altered sensations on the medial face of the leg, and suggested that this alteration was not related to injury of the IPBSN branch but rather, injury to the sartorius branch of the saphenous nerve (SBSN). This type of injury is thought to be related to the passage of the stripper. The historic incidence of IPBSN injury reported in ACLR surgeries was mostly BPTB graft harvest cases. Mochizuki et al. [12] described an interdigitating innervation of nerves supplying the upper anterior leg region with lots of variation, having the medial femoral cutaneous nerve and the SN as their roots, so that the boundary between them cannot be distinguished. They also described that tendon stripping may not always be the reason, as their findings are more in favour of vertical incision-related sensory disturbance. Their rationale is that stripping will cause trunk injury, which will result in widespread loss. This study throws a possibility of even more distal IPBSN branches, distal to the tibial tuberosity (apart from the commonly described two branches), which can lead to distal lateral leg (more of the sural nerve dermatome) sensory disturbance. We had five patients with a similar pattern of paraesthesia, but were included as trunk injury itself based on the lack of extensive evidence on these branches.

We were interested in finding out the exact reason for our IPBSN injuries, whether they were due to the portal incisions, vigorous skin retraction, or deep blunt dissection undermining the skin during tendon exploration, or blunt injury by the stripper during harvesting. But this requires more elaborate clinical and cadaveric studies with larger samples. By all means, we believe combined IPBSN and SBSN affection may be due to the SN trunk blunt trauma incited by the stripper while it negotiates across the hamstring, especially the gracilis, at or near the joint line. So, we could conclude that the isolated IPBSN injury was found to be highly unlikely following our harvest incision. So, the most probable way for it was the stripping, apart from the possibility of injury during the placement of the portal incision. The stripping of the tendon, in theory, can lead to trunk or branch injury, which can explain the different patterns of sensory disturbance we saw in our results.

## Limitation

This study has certain limitations that should be acknowledged. First, the non-randomised design may introduce selection bias; however, randomisation was not feasible as the decision to harvest the gracilis tendon was made intra-operatively based on objective graft adequacy criteria to ensure optimal reconstruction. Second, although all procedures were performed by a single experienced surgeon using a standardised technique to minimise variability, surgeon-dependent factors cannot be completely eliminated. Third, sensory outcomes were assessed using patient-reported symptoms rather than objective neurophysiological testing, which may have limited the precision of sensory deficit detection, although patient-perceived disturbance remains clinically most relevant. Additionally, the follow-up period was limited to six months; while this duration is considered sufficient to capture persistent sensory disturbances, a longer follow-up could provide further insight into late recovery patterns. Finally, this was a single-centre study, which may limit the generalisability of the findings.

## Conclusion

When harvesting hamstring grafts for ACL reconstruction, our study shows that the saphenous nerve is more vulnerable to damage, particularly when semitendinosus and gracilis are harvested together rather than semitendinosus alone. Our finding is anatomically plausible given the close relationship between the gracilis tendon and the saphenous nerve branches. While this association is statistically significant and consistent with prior anatomical and clinical observations, its clinical implications should be interpreted with caution. Most sensory disturbances were mild and did not translate into meaningful functional impairment, underscoring that the primary impact relates to donor-site morbidity rather than major limitation in daily activities. Nevertheless, even minor sensory symptoms may affect patient comfort and satisfaction, particularly in young, active individuals. These findings highlight the importance of judicious graft selection, favouring isolated semitendinosus harvest when graft adequacy permits, in order to minimise avoidable nerve-related complications without compromising reconstructive outcomes.

## Data Availability

This article has no associated data generated and/or analysed.
